# Frequency of gestational diabetes mellitus and the associated risk factors 

**DOI:** 10.12669/pjms.311.5617

**Published:** 2015

**Authors:** Nuriye Buyukkayaci Duman

**Affiliations:** Nuriye Buyukkayaci Duman, Assistant Professor, Hitit University School of Health, Corum, Turkey.

**Keywords:** Affecting Factors, Frequency, Gestational Diabetes Mellitus

## Abstract

**Objective:**

This prospective study was undertaken to assess the frequency of gestational diabetes mellitus (GDM) and the associated risk factors during pregnancy.

**Methods:**

The sample consisted of 650 pregnant women who had no known risk factors for GDM and were followed-up at the Outpatient Clinic for Pregnant Women, Corum State Hospital between March 2009 and June 2010. The data were expressed as percentage, arithmetic mean, standard deviation and chi-square test.

**Results:**

Of the 650 pregnant women, 45 were diagnosed with GDM during the study period (6.9%). A statistically significant correlation between GDM and advanced age, family history, and body mass index was found, while no significant correlations existed between GDM and the frequency of pregnancy, number of pregnancy, parity, and number of live births.

**Conclusion:**

Advanced age, high body mass index and family history of diabetes mellitus emerged as risk factors for GDM in our study.

## INTRODUCTION

Gestational diabetes mellitus (GDM) is a glucose tolerance disorder of any severity occurring for the first time or diagnosed during pregnancy.^1^^,^^2^ The reported global prevalence of GDM ranges between 1% and 14%.^3^^-^^7^ Diabetogenic effect of pregnancy is thought to play a role in the development of GDM where human placental lactogenic hormone (HPL) secreted from placenta during pregnancy results in insulin desensitization leading to physiological increases in blood glucose levels, particularly during the 2^nd^ and 3^rd^ trimesters.^8^ Also increase in the circulating levels of growth hormone, cortisone, estrogen and progesterone is considered to play a contributing role for the insulin resistance.^6^^,^^7^

Several other factors including maternal age, ethnicity, genetic disposition, polycystic ovary syndrome, hypertension and obesity have also been associated with the risk of GDM.^1^^,^^9^ GDM, if not managed properly, may lead to variety of complications, both for the mother and the baby, during and after the pregnancy.^10^^-^^12^ GDM is also associated with an increased risk of certain other conditions such as pre-eclampsia, polyhydramnios, fetal macrosomia, hyperbilirubinemia, hypoglycemia, hypocalcemia, polycythemia, mental retardation, birth trauma and neonatal mortality.^2^^,^^10^^-^^12^

Thus, early detection and treatment of GDM carries a significant importance for maternal and fetal health. Our study aimed at assessing the prevalence of this condition along with its associated risk factors, in a sample of pregnant women.

## METHODS

The sample population consisted of 650 pregnant women selected using a simple-random sampling scheme who attended to the Outpatient Clinic for Pregnant Women, Corum State Hospital, Turkey, between March 2009 and June 2010. The data were gathered using the Data Collection Form for Descriptive Characteristics of Diabetic Women, which was specifically designed by the members of the research team using previously published data.

***Data Collection Form For Descriptive Characteristics of Diabetic Women: ***The form included a total of 10 items that address certain socio-demographic, obstetric and diabetes-related characteristics (such as the age, educational status, profession, number of pregnancy, number of parity, time interval between two pregnancies, family history of diabetes, GDM status in the previous pregnancy, and the result of oral glucose tolerance test).

**Fig.1:**
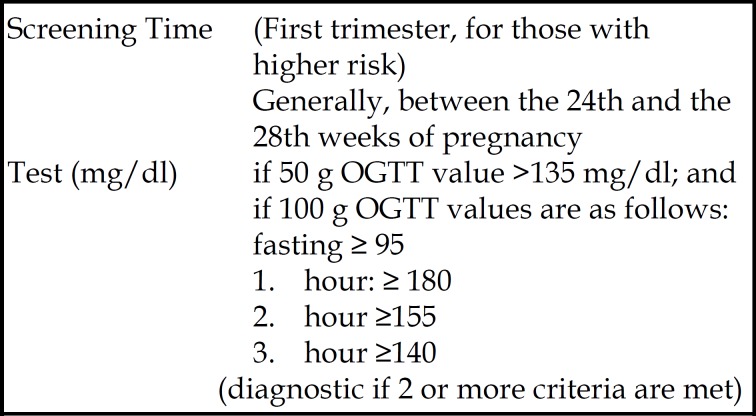
GDM criteria of ACOG (2001).

***Data Assessment: ***During the study period, the first 50 g oral glucose tolerance test (OGTT) was performed between 24 and 28 weeks of the pregnancy, and a second 100 g OGTT was administered to those with a glucose value of ≥ 135 mg/dl, according to the diagnostic criteria proposed by The American Congress of Obstetricians and Gynecologists (ACOG, 2001).^13^ Subjects with two or more test results higher than the normal range were considered to have GDM (Fig.1).

The data were analyzed with SPSS 17.0 statistical package program for Windows; and comparisons were done using chi-square test in computer environment.

***Ethical Considerations: ***The study protocol both for the pre-test and implementation phase was approved by the Institutional Ethics Committee, Corum State Hospital. All participants provided written informed consent following the provision of oral and written information on the purpose of the study.

## RESULTS

Approximately one-third (61.8%) of the participants were between 23 and 28 years of age and half (53.8%) had a primary school level of education. Nearly 70% of the cases were having their first pregnancy. Majority of the multigravida women were multiparous (75.0%). In addition, most of the women did not have a family history of diabetes (76.9%) (Table-I).

A diagnosis of diabetes was made in 45 out of 650 patients i.e. 6.9% of the participants. Variables that were found to be associated with a significantly increased risk of gestational diabetes included age, body mass index, family history of diabetes and past gestational diabetes (p< 0.05) (Table-II). Accordingly, proportion of patients with a diagnosis of GDM was 2.5%, 12.5%, and 75.0% in those between 23-28, 29-34, and 34-39 years of age, respectively. On the other hand, educational status, occupational status, number of pregnancy, and number of parity had no effect on the risk of GDM (p< 0.05) (Table-II).

## DISCUSSION

GDM is *defined* as any degree of glucose intolerance with onset or first recognition during pregnancy and it generally develops during the 2^nd^ and 3^rd^ trimester of the pregnancy, in conjunction with the diabetogenic effect of HPL secretion through placental blood circulation after 14^th^ week of pregnancy.^2^^,^^8^ In addition to this physiological effect, glucose intolerance and obesity also may play a role in the development GDM.^9^^,^^14^^,^^15^ In turn, GDM may increase the risk of a number of pregnancy-related complications such as spontaneous abortion and preeclampsia and the risk of fetal complications such as macrosomia, hyperbilirubinemia, polycythemia, and hypocalcemia, contributing to increased number of neonatal deaths and cesarean sections.^2^^,^^9^^,^^10^ Therefore GDM screening is important both for the maternal and fetal health. On the other hand, some experts hold the view that routine GDM screening may actually not be necessary due to the absence of solid data that show a high complication rate among women with GDM.^1^^,^^8^^,^^13^ In addition, opinions as to the timing of GDM screening differ. For instance, according to WHO all pregnant women should be screened for GDM between 24^th^ and 28^th^ week of pregnancy.^8^ On the other hand, ACOG (2001) and American Diabetes Association (ADA, 2004) recommend that high-risk pregnant women (age ≥ 25 years, obese, previous history of GDM, presence of a large fetus for gestational age, glucosuria and polycystic ovary) be screened in the first trimester while screening between the 24^th^ and 28^th^ weeks of pregnancy may be appropriate for the remaining pregnant women.^1^^,^^13^ If there are no known risk factors, ACOG (2001) also endorses the “no-screen” option.^13^ In our study site, high-risk pregnant women were screened in the first trimester as suggested by ACOG (2001) and ADA (2004) while others were screened between 24^th^ and 28^th^ weeks.^1^^,^^13^ With this screening schedule, 6.9% of the pregnant women participating in our study were found to have GDM.

**Table-I:** Demographic and clinical characteristics

Characteristics	N	%
Age (Years)	N:650	
23-28 29-34 34-39	402 200 48	61.8 30.8 7.4
Educational Status	N:650	
Primary School Secondary School High School and above	350 150 150	53.8 23.1 23.1
Occupation	N:650	
Yes No	250 400	38.5 61.5
Number of Pregnancies	N:650	
Primigravida Multigravida	450 200	69.2 30.8
Parity	n:200*	
Primipara Multipara	50 150	25.0 75.0
Family history of diabetes	N:650	
Yes No	150 500	23.1 76.9
GDM during the previous pregnancy	n:200*	
Yes No	15 185	7.5 92.5
Body Mass Index (BMI)	N:650	
underweight (13–18.4 kg/ m^2^) normal weight (18.5–24.9 kg/m^2^) overweight (25–29.9 kg/m^2 ^) obese (30–34.9 kg/m^2^)	80 400 120 50	12.3 61.5 18.5 7.7

* responded by more than one pregnant women.

**Table-II:** Distribution of GDM frequency by demographic and clinical characteristics

Characteristics	GDM (%)		%	Chi Square	p
	Yes	No			
Age (years)					
23-28 29-34 34-39	2.5 12.5 75.0	97.5 87.5 25.0	100.0 100.0 100.0	1.455	0.345*
Educational status					
Primary School Secondary School High School and above	10.5 13.6 11.4	89.5 96.4 88.6	100.0 100.0 100.0	8.001	0.678
Occupation					
Yes No	15.6 12.2	84.4 87.8	100.0 100.0	9.988	0.978
Number of pregnancies					
Primigravida Multigravida	13.5 10.9	86.5 89.1	100.0 100.0	7.624	0.645
Parity					
Primipara Multipara	12.3 13.5	87.7 86.5	100.0 100.0	5.456	0.554
Family history of diabetes					
Yes No	35.0 12.0	65.0 88.0	100.0 100.0	3.456	0.222*
GDM during the previous pregnancy					
Yes No	100.0 5.5	0.0 94.5	100.0 100.0	7.456	0.002*
Body Mass İndex (BMİ)					
under weight (13–18.4 kg/ m^2^) normal (18.5–24.9 kg/m^2^) overweight (25–29.9 kg/m^2 ^) obese (30–34.9 kg/m^2^)	2.5 10.0 50.0 75.0	97.5 90.0 50.0 25.0	100.0 100.0 100.0 100.0	1.799	0.234*

* P <0.05

The reported GDM incidence in the literature varies. The figure reported by ADA (2004) was 4% in 2004, while rates between 4.9% and 12.8% were reported among high-risk populations such as the native tribes and North Californian natives in the US.^1^ In a study by Brody et al. (2003) 1 to 6% of the pregnant women developed hyperglycemia and GDM, similar to our observations.^6^ However, the detected prevalence of GDM in our population, i.e. 6.9%, is somewhat higher than those previously reported in other Turkish populations, which range between 1 and 5%. These differences may probably be explained on the basis of the differences in study populations in terms of sample size or age. Literature data suggest that a number of factors including advanced age, obesity, genetic disposition and a previous history of GDM may increase the risk of GDM.^9^^,^^14^^-^^19^ Similar to these suggestions, age, BMI, family history of diabetes, and GDM in previous pregnancy emerged as important risk factors for GDM in our study with a statistically significant contribution of these factors (p<0.05) to the occurrence of GDM. Studies by Turgut et al. and Dunhbai et al. provide a clear support for the role of advanced age in the development of GDM.^18^^,^^19^ In the former study, other risk factors for GDM included a history of DM in the first-degree relatives and a history of GDM in previous pregnancy.^19^ Similarly, the studies by Di Cianni et al. and Kaya pointed out to advanced age and BMI as risk factors for GDM.^20^^,^^21^ Our findings are in agreement with these reports.

## CONCLUSIONS

As a result, it was seen in the study that diabetes developed among the 6.9% of women. It was noted that there was a positive correlation between age, family diabetes history and past gestational diabetes, and gestational diabetes. Based on the study findings; the following recommendations are made:

1. The women who are admitted to the follow-up polyclinics for pregnant women due to diagnosis and treatment should be provided with counseling about diabetogenic features of pregnancy and should be informed about the importance of screening tests.

2. Especially those who are aged over 35 and have diabetes family history and/or past pregnancy diabetes, high BMİ should be followed-up more closely in gestational diabetes. 

3. The future studies on GDM should be undertaken with bigger sample sizes through randomized controlled groups.

## Notes

***Note: ***This study was presented as an oral presentation in the 4^th^ World Congress on Diabetes and Metabolism, August 14-16, 2013 Chicago-North Shore, IL, USA.
